# Supplementary material for: Evaluation of the Synovial Effects of Biological and Targeted Synthetic DMARDs in Patients with Psoriatic Arthritis: A Systematic Literature Review and Meta-Analysis
Source: Int J Mol Sci. 2023 Mar 5;24(5):5006. doi: 10.3390/ijms24055006 (PMC10002880; doi:10.3390/ijms24055006)
Supplement: Supplementary file 1 [file ijms-24-05006-s001.zip › ijms-2191805-supplementary.pdf]

Supplementary Material

1.0 Materials and Methods

1.1 PICOs

P: Population: patients with psoriasis or psoriatic arthritis (PsA);

I: synovial biopsies (paired biopsies in longitudinal studies with systemic administration of bDMARDs or tsDMARDs approved for the systemic management of these conditions, *in vitro* studies with bDMARDs or tsDMARDs approved for the systemic management of these conditions);

C: any;

O: biomarkers modifications on synovial membrane biopsies; clinical response to systemic treatment;

S: Study design: systematic literature reviews and meta-analyses, randomized-controlled trials, non-controlled trials, diagnostic accuracy studies, cohort studies, cross-sectional studies, case-control studies, case series (>5), *in vitro* studies, congress proceedings and abstracts (2020-2021).

1.2 Inclusion criteria and Exclusion criteria:

Study design(s)

- Inclusion criteria: The SLR will include systematic literature reviews and meta-analyses, randomized-controlled trials, non-controlled trials, diagnostic accuracy studies, cohort studies, cross-sectional studies, case-control studies, case series (≥ 5), *in vitro* studies, congress proceedings and abstracts (2020-2021).

- Exclusion criteria: Excluded articles will include review articles, congress proceedings and abstracts (before 2019), duplicate publication, letters to the editor, case series (<5), case reports.

Main outcome(s)

- Inclusion criteria: Biological effects (e.g. biomarkers modifications) of b/tsDMARDs on target tissues (i.e. skin and synovium).
- Exclusion criteria: Studies not reporting biological effects of selected drugs.

Measures of effect

- Standardized mean difference of biomarkers variations.

Additional outcome(s)

Predictive power of biomarkers for therapeutical efficacy of systemic treatment.

1.3 Keywords and search strategies

Keywords

Psoriasis, psoriatic arthritis, biopsy, skin, joint, synovial membrane, in vitro techniques, TNF inhibitors, etanercept, adalimumab, infliximab, golimumab, certolizumab, tofacitinib, upadacitinib, apremilast, secukinumab, ixekizumab, risankizumab, brodalumab, ustekinumab, tildrakizumab, guselkumab, abatacept, bimekizumab.

Search strategies

MEDLINE (via Pubmed):

((“Arthritis, Psoriatic”[Mesh]) OR (psoria*[TW] AND arthriti*[TW]) OR (psoria*[TW] AND arthropath*[TW]) OR (psoria*[TW] AND poly-arthriti*[TW]) OR (psoria*[TW] AND oligoarthr*[TW]) OR (psoria*[TW] AND oligoarthr*[TW]) OR (psoria*[TW] AND oligo-arthr*[TW]) OR (psoria*[TW] AND rheumato*[TW]) OR (psoriasis[MeSH Terms]) OR (psoria*[All Fields]) OR (palmoplantar* pustulosis[All Fields]) OR (pustulosis palmaris et plantaris[All Fields]) OR (pustulosis[All Fields] AND palms[All fields] AND soles[All Fields]))

AND

(((“pathology”[MeSH Terms] OR “pathology”[MeSH Subheading] OR "biopsie"[All Fields] OR "biopsy"[MeSH Terms] OR "biopsy"[All Fields] OR "biopsied"[All Fields] OR "biopsies"[All Fields] OR "biopsy s"[All Fields] OR "biopsying"[All Fields] OR "biopsys"[All Fields])

AND

(“skin”[MeSH Terms] OR “joints”[MeSH Terms] OR "joints"[All Fields] OR "joint"[All Fields] OR "synovial membrane"[All Fields])) OR “punch biopsy”[All fields] OR “synovial biopsy”[All fields] OR "synovial membrane/pathology"[MeSH Terms] OR "synovial membrane/immunology"[MeSH Terms] OR "In Vitro Techniques"[Majr])

AND

("tnf inhibitors"[Text Word] OR "tnf inhibit*"[Text Word] OR "tumor necrosis factor inhibit*"[Text Word] OR "tumour necrosis factor inhibit*"[Text Word] OR "anti-tnf"[Text Word] OR "antitnf"[Text Word] OR "anti tumor necrosis factor*"[Text Word] OR "anti tumour necrosis factor*"[Text Word] OR "Etanercept"[MeSH Terms] OR "Adalimumab"[MeSH Terms] OR "Infliximab"[MeSH Terms] OR "Certolizumab Pegol"[MeSH Terms] OR "golimumab"[Supplementary Concept] OR "receptors, tumor necrosis factor"[MeSH Terms] OR "Tumor Necrosis Factors"[MeSH Terms] OR "antibodies,

monoclonal"[MeSH Terms] OR "enbrel"[Title/Abstract] OR "benepali"[Title/Abstract] OR "erelzi"[Title/Abstract] OR "humira"[Title/Abstract] OR "amjevita"[Title/Abstract] OR "amgevita"[Title/Abstract] OR "imraldi"[Title/Abstract] OR "hyrimoz"[Title/Abstract] OR "hefiya"[Title/Abstract] OR "halimatoz"[Title/Abstract] OR "solymbic"[Title/Abstract] OR "trudexa"[Title/Abstract] OR "cyltezo"[Title/Abstract] OR "remicade"[Title/Abstract] OR "inflectra"[Title/Abstract] OR "remsima"[Title/Abstract] OR "ixifi"[Title/Abstract] OR "renflexis"[Title/Abstract] OR "flixabi"[Title/Abstract] OR "zessly"[Title/Abstract] OR "cimzia"[Title/Abstract] OR "simponi"[Title/Abstract] OR "Etanercept"[Text Word] OR "Adalimumab"[Text Word] OR "Infliximab"[Text Word] OR "certolizumab"[Text Word] OR "golimumab"[Text Word] OR "tofacitinib"[Supplementary Concept] OR "tofacitinib"[All Fields] OR "tofacitinib's"[All Fields] OR "xeljanz"[All Fields] OR "upadacitinib"[Supplementary Concept] OR "upadacitinib"[All Fields] OR "rinvoq"[All Fields] OR "apremilast"[Supplementary Concept] OR "apremilast"[All Fields] OR "otezla"[All Fields] OR "secukinumab"[Supplementary Concept] OR "secukinumab"[All Fields] OR "cosentyx"[All Fields] OR "ixekizumab"[Supplementary Concept] OR "ixekizumab"[All Fields] OR "taltz"[All Fields] OR "risankizumab"[Supplementary Concept] OR "risankizumab"[All Fields] OR "skyrizi"[All Fields] OR "brodalumab"[Supplementary Concept] OR "brodalumab"[All Fields] OR "kyntheum"[All Fields] OR "ustekinumab"[MeSH Terms] OR "ustekinumab"[All Fields] OR "stelara"[All Fields] OR "tildrakizumab"[Supplementary Concept] OR "tildrakizumab"[All Fields] OR "ilumetri"[All Fields] OR "guselkumab"[Supplementary Concept] OR "guselkumab"[All Fields] OR "tremfya"[All Fields] OR "abatacept"[MeSH Terms] OR "abatacept"[All Fields] OR "orencia"[All Fields] OR "Receptors, Interleukin-17/antagonists and inhibitors"[MeSH] OR "Interleukin-23/antagonists and inhibitors"[MeSH] OR "Janus Kinase Inhibitors"[MeSH] OR "bimekizumab"[Supplementary Concept] OR "bimekizumab"[All Fields])
NOT ("animals"[MeSH Terms] NOT "humans"[MeSH Terms])

Embase:
(('psoriatic arthritis'/exp) OR (psoria* AND arthriti*) OR (psoria* AND arthropath*) OR (psoria* AND polyarthriti*) OR (psoria* AND 'poly arthriti*') OR (psoria* AND oligoarthr*) OR (psoria* AND 'oligo arthr*') OR (psoria* AND rheumato*) OR ('psoriasis'/exp) OR ('psoriasis' OR psoria*:ti,ab) OR (palmoplantar* AND pustulosis:ti,ab) OR (('pustulosis'/exp) OR ((pustulosis AND palmaris AND et AND plantaris):ti,ab)) OR (('pustulosis'/exp) OR ((pustulosis AND palms AND soles):ti,ab)))
AND

((('pathology'/exp OR 'molecular pathology'/exp OR 'biopsy'/exp OR 'rebiopsy'/exp) AND ('skin'/exp OR 'cutis' OR 'derma' OR 'human skin' OR 'skin' OR 'skin layer' OR 'joint' OR 'joints' OR 'synovial joint' OR 'joint'/exp OR 'synovium'/exp OR 'membrana synovialis' OR 'membrane, synovial' OR 'synovia membrane' OR 'synovia tissue' OR 'synovial lining' OR 'synovial membrane' OR 'synovial tissue' OR 'synovialis' OR 'synovium' OR 'in vitro study'/exp) OR 'ex vivo study'/exp) OR 'punch biopsy'/exp OR 'biopsy, punch' OR 'micropuncture' OR 'punch biopsy' OR 'punction' OR 'joint biopsy'/exp OR 'knee biopsy' OR 'synovia biopsy' OR 'synovial biopsy' OR 'synovium biopsy')

AND

(('tumor necrosis factor inhibitor'/exp) OR ('tnf alpha inhibitor') OR ('tnf inhibitor') OR ('anti tnf agent') OR ('anti tnf alpha agent') OR ('anti tumor necrosis factor agent') OR ('anti tumour necrosis factor agent') OR ('tumor necrosis factor alpha inhibitor') OR ('tumor necrosis factor inhibitor') OR ('tumor necrosis factor inhibitors') OR ('tumour necrosis factor alpha inhibitor') OR ('tumour necrosis factor inhibitor') OR ('etanercept'/exp) OR ('benepali') OR ('embrel') OR ('enbrel') OR ('erelzi') OR ('etanercept') OR ('adalimumab'/exp) OR ('amgevita') OR ('amjevita') OR ('cyltezo') OR ('hefiya') OR ('humira') OR ('hyrimoz') OR ('imraldi') OR ('yuflyma') OR ('adalimumab') OR ('trudexa') OR ('solymbic') OR ('halimatoz') OR ('infliximab'/exp) OR ('flixabi') OR ('inflectra') OR ('infliximab') OR ('ixifi') OR ('remicade') OR ('remsima') OR ('renflexis') OR ('zessly') OR ('certolizumab pegol'/exp) OR ('certolizumab pegol') OR ('cimzia') OR ('golimumab'/exp) OR ('golimumab') OR ('simponi') OR ('tofacitinib'/exp) OR ('tofacitinib') OR ('tofacitinib citrate') OR ('xeljanz') OR ('upadacitinib'/exp) OR ('rinvoq') OR ('upadacitinib') OR ('apremilast'/exp) OR ('apremilast') OR ('otezla') OR ('secukinumab'/exp) OR ('cosentyx') OR ('secukinumab') OR ('ixekizumab'/exp) OR ('ixekizumab') OR ('taltz') OR ('risankizumab'/exp) OR ('risankizumab') OR ('skyrizi') OR ('brodalumab'/exp) OR ('brodalumab') OR ('kyntheum') OR ('ustekinumab'/exp) OR ('stelara') OR ('ustekinumab') OR ('tildrakizumab'/exp) OR ('ilumetri') OR ('ilumya') OR ('tildrakizumab') OR ('guselkumab'/exp) OR ('guselkumab') OR ('tremfya') OR (‘abatacept’) OR (‘orencia’) OR ('interleukin 17 antibody') OR ('interleukin 23 antibody') OR (‘interleukin 17 inhibitor’) OR (‘interleukin 23 inhibitor’) OR (‘Janus kinase inhibitor*’) OR (‘bimekizumab’) OR ('bimekizumab'/exp) OR ('bimzelx'))

Scopus:

(TITLE-ABS-KEY (psoriatic AND arthritis) OR TITLE-ABS-KEY (psoriasis))

AND

(TITLE-ABS-KEY (joint AND biopsy) OR TITLE-ABS-KEY (synovial AND biopsy) OR TITLE-ABS-KEY (skin AND biopsy) OR TITLE-ABS-KEY (in AND vitro AND study) OR TITLE-ABS-KEY (ex AND vivo AND study) OR TITLE-ABS-KEY (synovial AND membrane AND pathology) OR TITLE-ABS-KEY (synovial AND membrane AND metabolism))

AND

(TITLE-ABS-KEY (tofacitinib) OR TITLE-ABS-KEY (upadacitinib) OR TITLE-ABS-KEY (apremilast) OR TITLE-ABS-KEY (etanercept) OR TITLE-ABS-KEY (adalimumab) OR TITLE-ABS-KEY (infliximab) OR TITLE-ABS-KEY (certolizumab) OR TITLE-ABS-KEY (golimumab) OR TITLE-ABS-KEY (secukinumab) OR TITLE-ABS-KEY (ixekizumab) OR TITLE-ABS-KEY (risankizumab) OR TITLE-ABS-KEY (brodalumab) OR TITLE-ABS-KEY (ustekinumab) OR TITLE-ABS-KEY (tildrakizumab) OR TITLE-ABS-KEY (guselkumab) OR TITLE-ABS-KEY (abatacept) OR TITLE-ABS-KEY (il-17 AND inhibitor) OR TITLE-ABS-KEY (il-23 AND inhibitor) OR TITLE-ABS-KEY (il-23 AND antibody) OR TITLE-ABS-KEY (il-17 AND antibody) OR TITLE-ABS-KEY (jak AND inhibitor) OR TITLE-ABS-KEY (bimekizumab))

Cochrane Library:

"psoriasis arthropathica" in All Text OR "psoriases" in All Text OR "psoriasis diffusa" in All Text OR "psoriasis guttata" in All Text AND "biopsy" in All Text

2.0 Supplementary results

2.1 Sensitivity analysis

2.1.1 PsA sensitivity analysis of CD3 meta-analysis

```
                              SMD        95%-CI           %W(random)
Goedkoop 2004 infliximab   -0.7012 [-1.6531;  0.2507]       33.0
Kruithof 2005 etanercept   -0.8200 [-1.4653; -0.1747]        0.0
Kruithof 2005 infliximab   -1.1469 [-2.0928; -0.2011]        0.0
Pontifex 2011 etanercept   -1.1389 [-2.0015; -0.2763]       40.2
Szentpetery 2017 abatacept -0.3259 [-1.3805;  0.7286]       26.9

Number of studies combined: k = 3
Number of observations: o = 116

                         SMD           95%-CI        z      p-value
Random effects model -0.7761 [-1.3227; -0.2295] -2.78  0.0054

Quantifying heterogeneity:
 tau^2 = 0 [0.0000; 6.2997]; tau = 0 [0.0000; 2.5099]
 I^2 = 0.0% [0.0%; 89.6%]; H = 1.00 [1.00; 3.10]

Test of heterogeneity:
    Q d.f. p-value
 1.40    2  0.4958

Details on meta-analytical method:
- Inverse variance method
- Restricted maximum-likelihood estimator for tau^2
- Q-profile method for confidence interval of tau^2 and tau
- Cohen's d (standardised mean difference)
```

2.1.2 TNFi sensitivity analysis of CD3 meta-analysis

```
                              SMD     95%-CI              %W(random)
Goedkoop 2004 infliximab   -0.7012 [-1.6531;  0.2507]       18.5
Kruithof 2005 etanercept   -0.8200 [-1.4653; -0.1747]       40.2
Kruithof 2005 infliximab   -1.1469 [-2.0928; -0.2011]       18.7
Pontifex 2011 etanercept   -1.1389 [-2.0015; -0.2763]       22.5
Szentpetery 2017 abatacept -0.3259 [-1.3805;  0.7286]        0.0

Number of studies combined: k = 4
Number of observations: o = 116

                         SMD           95%-CI       z       p-value
Random effects model -0.9311 [-1.3405; -0.5217] -4.46 < 0.0001
```

Quantifying heterogeneity:
tau^2 = 0 [0.0000; 0.5014]; tau = 0 [0.0000; 0.7081]
I^2 = 0.0% [0.0%; 84.7%]; H = 1.00 [1.00; 2.56]

Test of heterogeneity:

Q	d.f.	p-value
0.76	3	0.8588

Details on meta-an alytical method:
- Inverse variance method
- Restricted maximum-likelihood estimator for tau^2
- Q-profile method for confidence interval of tau^2 and tau
- Cohen's d (standardised mean difference)

2.1.3 Semiquantitative score sensitivity analysis of CD3 meta-analysis (using MD)

	MD	95%-CI	%W(random)
Goedkoop 2004 infliximab	-69.0000	[-159.9221; 21.9221]	0.0
Kruithof 2005 etanercept	-0.6062	[-1.0645; -0.1480]	64.3
Kruithof 2005 infliximab	-0.9500	[-1.6760; -0.2240]	25.6
Pontifex 2011 etanercept	-278.2708	[-473.7781; -82.7635]	0.0
Szentpetery 2017 abatacept	-0.3600	[-1.5172; 0.7972]	10.1

Number of studies combined: k = 3
Number of observations: o = 116

	MD	95%-CI	z	p-value
Random effects model	-0.6695	[-1.0369; -0.3020]	-3.57	0.0004

Quantifying heterogeneity:
tau^2 = 0 [0.0000; 3.2399]; tau = 0 [0.0000; 1.8000]
I^2 = 0.0% [0.0%; 89.6%]; H = 1.00 [1.00; 3.10]

Test of heterogeneity:

Q	d.f.	p-value
0.92	2	0.6308

Details on meta-analytical method:
- Inverse variance method
- Restricted maximum-likelihood estimator for tau^2
- Q-profile method for confidence interval of tau^2 and tau

2.1.4 PsA sensitivity analysis of CD68 meta-analysis

	SMD	95%-CI	%W(random)
Goedkoop 2004 infliximab	-0.7253	[-1.6791; 0.2285]	32.5
Pontifex 2011 etanercept	-0.0719	[-0.8723; 0.7285]	37.7
Cañete Juan D. 2004 infliximab	-1.4796	[-2.5223; -0.4369]	29.8

Kruithof 2005 etanercept   -0.7096 [-1.3487; -0.0706]   0.0
Kruithof 2005 infliximab   -1.0825 [-2.0210; -0.1439]   0.0

Number of studies combined: k = 3
Number of observations: o = 120

	SMD	95%-CI	z	p-value
Random effects model	-0.7040	[-1.5029; 0.0949]	-1.73	0.0841

Quantifying heterogeneity:
tau^2 = 0.2742 [0.0000; 19.3727]; tau = 0.5237 [0.0000; 4.4014]
I^2 = 55.1% [0.0%; 87.2%]; H = 1.49 [1.00; 2.79]

Test of heterogeneity:

Q	d.f.	p-value
4.46	2	0.1077

Details on meta-analytical method:
- Inverse variance method
- Restricted maximum-likelihood estimator for tau^2
- Q-profile method for confidence interval of tau^2 and tau
- Cohen's d (standardised mean difference)

2.2 Publication bias

2.2.1 Meta-analysis CD3 variation in response of bDMARDs
Eggers' test of the intercept

intercept	95% CI	t	p
0.71	-3.04 - 4.45	0.37	0.74

Eggers' test does not indicate the presence of funnel plot asymmetry.

2.2.2 Meta-analysis CD68sl variation in response of bDMARDs
Eggers' test of the intercept

intercept	95% CI	t	p
-2.76	-8.11 - 2.59	-1.01	0.39

Eggers' test does not indicate the presence of funnel plot asymmetry.

3.0 Supplementary Figures and Tables

Supplementary Table S1. Sensitivity analyses

	Outcome	N	SMD or MD* [95%CI]	I^2	p value
PsA studies	CD3	28	-0.78 [-1.32; -0.23]	0.0%	0.0054
TNFi studies	CD3	56	-0.93 [-1.34; -0.52]	0.0%	< 0.0001
Semiquantitative score studies	CD3	37	-0.67 [-1.04; -0.30]	0.0%	0.0004
PsA studies	CD68	30	-0.70 [-1.5; 0.09]	55.1%	0.0841

Abbreviations: PsA: psoriatic arthritis, TNFi: tumour necrosis factor inhibitors, SMD: standardised mean difference, MD: mean difference, 95%CI: 95% confidence interval.

Supplementary Figure S1. Funnel plot of meta-analysis CD3 variation in response of bDMARDs.

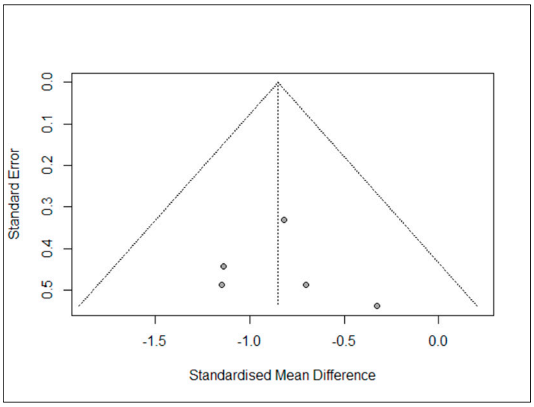

Supplementary Figure S2. Funnel plot of meta-analysis CD68sl variation in response of bDMARDs.

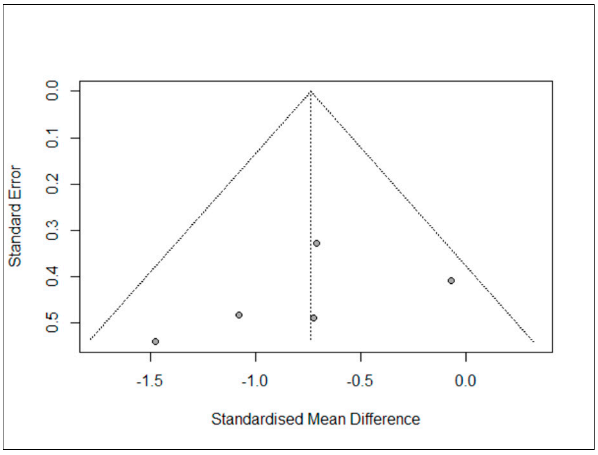

Supplementary Table S2. PRISMA 2020 Checklist [1]

Section and Topic	Item #	Checklist item	Location where item is reported
**TITLE**			
Title	1	Identify the report as a systematic review.	1
**ABSTRACT**			
Abstract	2	See the PRISMA 2020 for Abstracts checklist.	1
**INTRODUCTION**			
Rationale	3	Describe the rationale for the review in the context of existing knowledge.	2
Objectives	4	Provide an explicit statement of the objective(s) or question(s) the review addresses.	2
**METHODS**			
Eligibility criteria	5	Specify the inclusion and exclusion criteria for the review and how studies were grouped for the syntheses.	2-3; SM 2
Information sources	6	Specify all databases, registers, websites, organisations, reference lists and other sources searched or consulted to identify studies. Specify the date when each source was last searched or consulted.	3; SM 3-6
Search strategy	7	Present the full search strategies for all databases, registers and websites, including any filters and limits used.	SM 3-6
Selection process	8	Specify the methods used to decide whether a study met the inclusion criteria of the review, including how many reviewers screened each record and each report retrieved, whether they worked independently, and if applicable, details of automation tools used in the process.	2-3, SM 1-2
Data collection process	9	Specify the methods used to collect data from reports, including how many reviewers collected data from each report, whether they worked independently, any processes for obtaining or confirming data from study investigators, and if applicable, details of automation tools used in the process.	3
Data items	10a	List and define all outcomes for which data were sought. Specify whether all results that were compatible with each outcome domain in each study were sought (e.g. for all measures, time points, analyses), and if not, the methods used to decide which results to collect.	2-3, SM 1-2
	10b	List and define all other variables for which data were sought (e.g. participant and intervention characteristics, funding sources). Describe any assumptions made about any missing or unclear information.	3
Study risk of bias assessment	11	Specify the methods used to assess risk of bias in the included studies, including details of the tool(s) used, how many reviewers assessed each study and whether they worked independently, and if applicable, details of automation tools used in the process.	3
Effect measures	12	Specify for each outcome the effect measure(s) (e.g. risk ratio, mean difference) used in the synthesis or presentation of results.	3
Synthesis methods	13a	Describe the processes used to decide which studies were eligible for each synthesis (e.g. tabulating the study intervention characteristics and comparing against the planned groups for each synthesis (item #5)).	3
	13b	Describe any methods required to prepare the data for presentation or synthesis, such as handling of missing summary statistics, or data conversions.	3
	13c	Describe any methods used to tabulate or visually display results of individual studies and syntheses.	3
	13d	Describe any methods used to synthesize results and provide a rationale for the choice(s). If meta-analysis was performed, describe the model(s),	3

Section and Topic	Item #	Checklist item	Location where item is reported
		method(s) to identify the presence and extent of statistical heterogeneity, and software package(s) used.	
	13e	Describe any methods used to explore possible causes of heterogeneity among study results (e.g. subgroup analysis, meta-regression).	3
	13f	Describe any sensitivity analyses conducted to assess robustness of the synthesized results.	3
Reporting bias assessment	14	Describe any methods used to assess risk of bias due to missing results in a synthesis (arising from reporting biases).	N.A.
Certainty assessment	15	Describe any methods used to assess certainty (or confidence) in the body of evidence for an outcome.	3
**RESULTS**			
Study selection	16a	Describe the results of the search and selection process, from the number of records identified in the search to the number of studies included in the review, ideally using a flow diagram.	4
	16b	Cite studies that might appear to meet the inclusion criteria, but which were excluded, and explain why they were excluded.	N.A.
Study characteristics	17	Cite each included study and present its characteristics.	4-13
Risk of bias in studies	18	Present assessments of risk of bias for each included study.	9
Results of individual studies	19	For all outcomes, present, for each study: (a) summary statistics for each group (where appropriate) and (b) an effect estimate and its precision (e.g. confidence/credible interval), ideally using structured tables or plots.	6-8
Results of syntheses	20a	For each synthesis, briefly summarise the characteristics and risk of bias among contributing studies.	N.A.
	20b	Present results of all statistical syntheses conducted. If meta-analysis was done, present for each the summary estimate and its precision (e.g. confidence/credible interval) and measures of statistical heterogeneity. If comparing groups, describe the direction of the effect.	4-13; SM 7-11
	20c	Present results of all investigations of possible causes of heterogeneity among study results.	SM 11
	20d	Present results of all sensitivity analyses conducted to assess the robustness of the synthesized results.	11-12; SM 7-10
Reporting biases	21	Present assessments of risk of bias due to missing results (arising from reporting biases) for each synthesis assessed.	N.A.
Certainty of evidence	22	Present assessments of certainty (or confidence) in the body of evidence for each outcome assessed.	N.A.
**DISCUSSION**			
Discussion	23a	Provide a general interpretation of the results in the context of other evidence.	13-16
	23b	Discuss any limitations of the evidence included in the review.	16
	23c	Discuss any limitations of the review processes used.	16

Section and Topic	Item #	Checklist item	Location where item is reported
	23d	Discuss implications of the results for practice, policy, and future research.	13-16
**OTHER INFORMATION**			
Registration and protocol	24a	Provide registration information for the review, including register name and registration number, or state that the review was not registered.	2
	24b	Indicate where the review protocol can be accessed, or state that a protocol was not prepared.	2-3
	24c	Describe and explain any amendments to information provided at registration or in the protocol.	N.A.
Support	25	Describe sources of financial or non-financial support for the review, and the role of the funders or sponsors in the review.	16
Competing interests	26	Declare any competing interests of review authors.	16
Availability of data, code and other materials	27	Report which of the following are publicly available and where they can be found: template data collection forms; data extracted from included studies; data used for all analyses; analytic code; any other materials used in the review.	16

Abbreviations: SM: Supplementary Material, N.A.: not applicable. For more information, visit: http://www.prisma-statement.org/

Bibliography

1. Page, M.J.; McKenzie, J.E.; Bossuyt, P.M.; Boutron, I.; Hoffmann, T.C.; Mulrow, C.D.; Shamseer, L.; Tetzlaff, J.M.; Akl, E.A.; Brennan, S.E.; et al. The PRISMA 2020 Statement: An Updated Guideline for Reporting Systematic Reviews. *BMJ* **2021**, *372*, n71, doi:10.1136/bmj.n71.
